# Do Probiotics During In-Hospital Antibiotic Treatment Prevent Colonization of Gut Microbiota With Multi-Drug-Resistant Bacteria? A Randomized Placebo-Controlled Trial Comparing *Saccharomyces* to a Mixture of *Lactobacillus, Bifidobacterium*, and *Saccharomyces*

**DOI:** 10.3389/fpubh.2020.578089

**Published:** 2021-03-08

**Authors:** Grégoire Wieërs, Valérie Verbelen, Mieke Van Den Driessche, Ekaterina Melnik, Greet Vanheule, Jean-Christophe Marot, Patrice D. Cani

**Affiliations:** ^1^Service of Internal Medicine, Clinique Saint Pierre, Ottignies-Louvain-la-Neuve, Belgium; ^2^Service of Microbiology, Clinique Saint Pierre, Ottignies-Louvain-la-Neuve, Belgium; ^3^Metagenics Europe, Oostende, Belgium; ^4^WELBIO - Walloon Excellence in Life Sciences and BIOtechnology, Louvain Drug Research Institute, Metabolism and Nutrition Research Group, UCLouvain, Université catholique de Louvain, Brussels, Belgium

**Keywords:** probiotics, microbiota, antibiotic resistance, prevention, extended-spectrum beta-lactamase, AmpC & [beta]-lactamase, pseudomonas, clinics and hospitals

## Abstract

**Objective:** Most infections with *Enterobacteriaceae* producing AmpC β-lactamase (AmpC)-, extended-spectrum β-lactamase (ESBL)-, and carbapenemase-producing bacteria, vancomycin-resistant *Enterococcus* as well as naturally resistant non-fermenting bacteria such as *Pseudomonas aeruginosa*, are related to a prior colonization of the gut microbiota. The objective of this study was to determine whether treatment with probiotics during an antibiotic treatment could prevent the colonization of the gut microbiota with multi-drug resistant bacteria.

**Method:** In total, 120 patients treated for 10 days with amoxicillin-clavulanate antibiotics were included in a randomized, placebo-controlled, double-blinded trial, comparing the effects of a 30 days treatment with placebo *Saccharomyces boulardii* CNCM I-745® and a probiotic mixture containing *Saccharomyces boulardii, Lactobacillus acidophilus* NCFM, *Lactobacillus paracasei* Lpc-37, *Bifidobacterium lactis* Bl-04, and *Bifidobacterium lactis* Bi-07 (Bactiol duo®). Study treatment was initiated within 48 h of the antibiotic being initiated. Most of the patients included were elderly with a mean age of 78 years old with multiple comorbidities. Stools were collected at the time of inclusion in the trial, at the end of the antibiotic treatment, and the end of the study treatment. These were cultured on selective antibiotic media.

**Results:** Treatment with the probiotic mixture led to a significant decline in colonization with *Pseudomonas* after antibiotic treatment from 25 to 8.3% (*p* = 0.041). Colonization with AmpC-producing enterobacteria was transiently increased after the antibiotic treatment (*p* = 0.027) and declined after the probiotic intervention (p= 0.041). No significant changes were observed in the placebo and *Saccharomyces* groups. Up to 2 years after the trial, no infection with ESBL-producing bacteria was observed in the probiotic mixture group.

**Conclusion:** The association of *Saccharomyces boulardii* with specific strains of *Lactobacillus* and *Bifidobacterium* influences antibiotic treatment by counteracting the colonization of the colon microbiota with antibiotic-resistant pathogens.

## Introduction

Infections with multi-drug resistant bacteria (MDR) such as extended-spectrum β-lactamase (ESBL)-, AmpC β-lactamase (AmpC)-, and carbapenemase-producing Enterobacteria (CPE), vancomycin-resistant *Enterococcus* (VRE), or naturally resistant non-fermenting bacteria such as *Pseudomonas aeruginosa* are associated with a high mortality rate (1–3). The risk of developing an infection with MDR bacteria is relative to the abundance of fecal MDR (2, 4–6). Such colonization is also known as the resistome, which is associated with a long stay in a healthcare setting and multiple antibiotic treatments. In clinics, infection-control teams evaluate fecal colonization with MDR bacteria by examining the presence of bacteria growing *in vitro* on selective media.

The development of new antibiotics for active infection with MDR bacteria is very slow, highlighting the need for alternative strategies to prevent the spread of antibiotic-resistant bacteria. Among these strategies, the effectiveness of reducing colonization of the intestinal microbiota by MDR bacteria using probiotics is being supported by *in vitro* and clinical observations (7, 8). The use of probiotic strains such as *Lactiplantibacillus plantarum or Limosilactobacillus fermentum* (formerly named *Lactobacillus plantarum* or *Lactobacillus fermentum*) was associated with a reduction in colonization with naturally resistant pathogens, such as *Acinetobacter baumannii, Pseudomonas aeruginosa*, and *Candida albicans* (9, 10). Patients colonized with ESBL-producing *Enterobacteriaceae* were treated with a mixture of eight viable bacterial strains (Vivomix®) at a dose of 9.10^11^ twice daily for 2 month. They experienced a 2.5-fold decrease in bacterial colonization 1 year later (11). Additionally, the risk of colonization of the gut microbiota with VRE in patients with hematological malignancies is low in the presence of the genus Barnesiella in the Bacteroidetes group, following treatment with *L. paracasei* (8, 12–14).

Nevertheless, the use of probiotics is not a one-size-fits-all method, and probiotic strain specificities impact their ability to eradicate the resistome. This study aims to determine the clinical conditions in which probiotic treatment could decrease the burden of antibiotic resistance as defined by clinical standards.

## Patients and Methods

**Figure 1:**
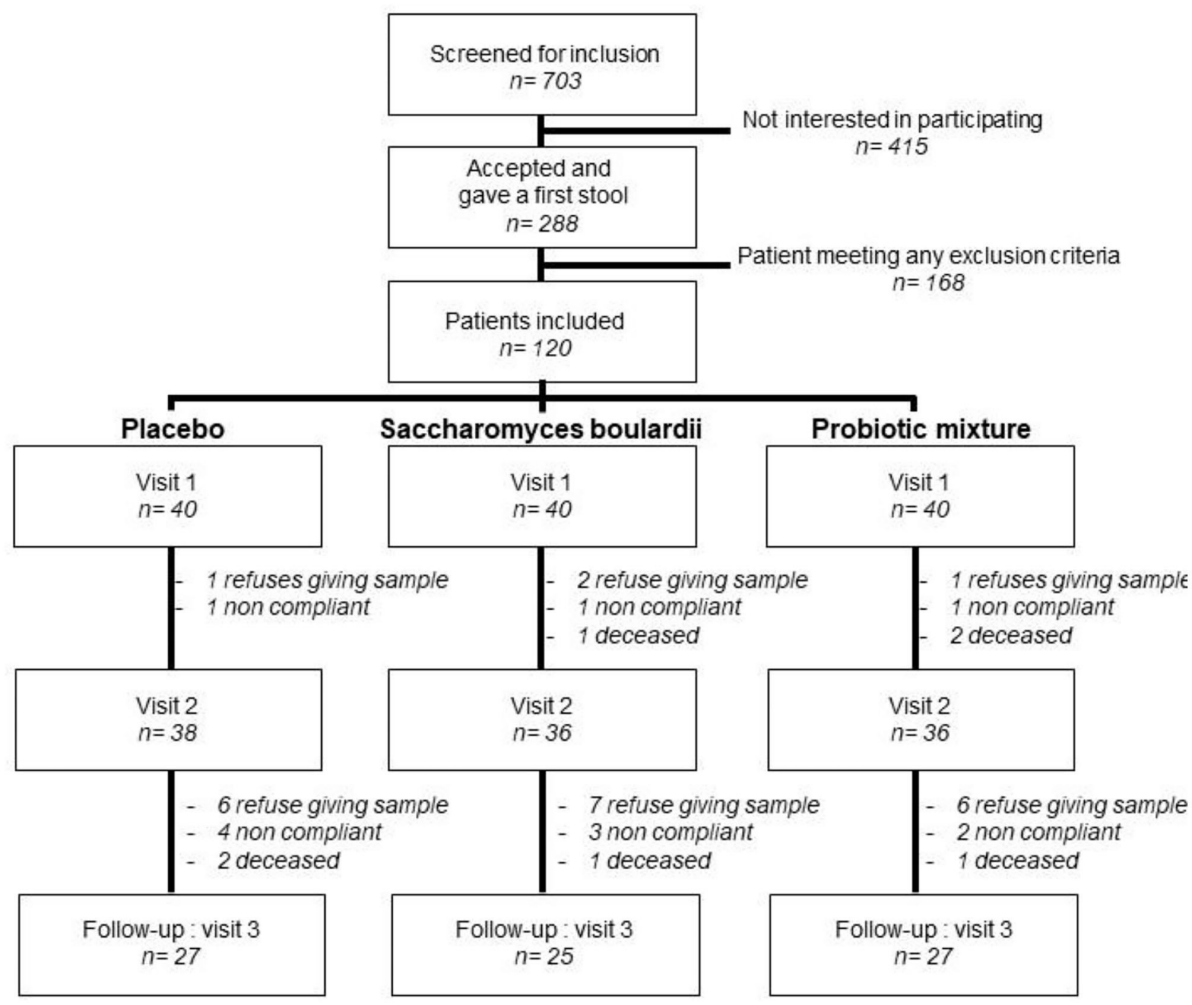
Randomized controlled trial flow chart. Ten patients were lost before collection of the stool sample at visit 2: Four refused to give the sample, three were not compliant with the treatment, and three died. Thirty-two patients were lost at visit 3: 19 refused to give the sample, nine were not compliant with the treatment, and four died.

This study was a double-blind, placebo-controlled, randomized trial. Infected patients treated with the antibiotic amoxicillin-clavulanate were randomly assigned to one of the three parallel groups in 1:1:1 ratio to receive treatment with a probiotic mixture, *Saccharomyces*, or placebo (Figure 1). The exclusion criteria were as follows: age below 18 years; pregnancy; breastfeeding; the average number of well-formed bowel movements more than three per day or fewer than three per week; participation in a clinical research trial 30 days prior to randomization; regular use of pro- and/or prebiotics; unstable medical condition; a history of chronic gastrointestinal disorders including irritable bowel syndrome, colitis, and Crohn's disease; allergy or sensitivity to test product ingredients or antibiotics; dialysis; deglutition abnormalities prohibiting or preventing normal oral intake; and treatment with other antibiotics at the time of randomization.

The patients were selected from the internal medicine and geriatrics wards at Clinique Saint Pierre in Ottignies, Belgium. They were hospitalized because of infectious diseases and treated empirically with amoxicillin 4,000 mg/clavulanate at 800 mg divided into four doses and administrated by the intravenous route, and amoxicillin at 2,625 mg/clavulanate at 375 mg divided into three doses and administrated by the oral route for a total of 10 days.

### Intervention

The patients were randomly assigned a probiotic mixture, *Saccharomyces boulardii*, or placebo. The probiotic mixture contained *Saccharomyces boulardii* at 6 × 10^9^ CFU/capsule; *Lactobacillus acidophilus* NCFM at 2 × 10^9^ CFU/capsule; *Bifidobacterium animalis* subsp. lactis Bi-07 at 2 × 10^9^ CFU/capsule; *Bifidobacterium animalis* subsp. lactis Bl-04 at 2 × 10^9^ CFU/capsule; *Lacticaseibacillus paracasei* Lpc-37 (formerly *Lactobacillus paracasei*) at 2 × 10^9^ CFU/capsule; blackberry fruit and leaf at 50 mg, cholecalciferol at 1.25 μg; and microcrystalline cellulose (40 mg), silicon dioxide (4 mg), and magnesium stearate excipients (4 mg) (Bactiol Duo®, Metagenics Europe). The *Saccharomyces* product contained *Saccharomyces boulardii* at 6 × 10^9^ CFU/capsule; mannitol (60 mg), magnesium stearate (6.7 mg), and silicon dioxide (3 mg) excipients. The placebo study product contained microcrystalline cellulose, dibasic anhydrous calcium phosphate, silicon dioxide, and magnesium stearate. The investigational product was labeled and coded according to the local regulatory guidelines.

Patients were instructed to take one capsule two times a day during a meal: once at breakfast and once at dinner. A nurse controlled the administration of the study product during hospitalization. The treatment continued at home up to 30 days of treatment with the study product. To monitor adherence at home, patients were instructed to return leftover capsules and/or empty boxes at the end of the study. Patients were also asked to complete a questionnaire on treatment compliance and tolerance.

The primary endpoint was the presence of positive fecal cultures with ESBL- or AmpC-producing bacteria, CPE, VRE, or naturally resistant non-fermenting bacteria after antibiotic treatment and after the study treatment.

A sample size of 40 subjects per group was required to detect clinically significant differences of 10% at 80% power, with 15% difference in statistical methods, allowing for a 20% attrition rate (15).

### Randomization and Blinding

For participant allocation, a computer-generated list of random numbers was created using a blocked randomization sequence with a random block size of two or three (R version 3.2.0 blockr and, R Core Team, 2019). Both study products and placebo products were filled in opaque capsules of identical appearance. They were prepacked in boxes and numbered according to the randomization schedule. Each patient was assigned an order number and received the capsule from the corresponding prepacked box. Treatment allocation depended on a random number list prepared by an investigator with no clinical involvement in the trial. The clinical investigators were responsible for dispensing the study product to the subjects as per the randomization schedule after obtaining the patients' consent. The patients and staff involved in product dispensing, visit assessment, the conduct of the study, and monitoring and analysis of data remained blinded for the duration of the study.

### Stool Analysis

A stool sample was collected in a sterile container at the time of inclusion in the trial on: visit 1, on average 42.4 ± 28.1 h after the first dose of antibiotics, at the end of the antibiotic treatment; visit 2, on average 11.6 ± 5.0 days after the first dose of antibiotics, and study treatment, and 1 to 3 months after discharge from the hospital; and visit 3, on average 60.8 ± 53.9 days after the first dose of antibiotics. All visits above are expressed as mean ± standard deviation. Samples were inoculated within 2 h of collection with a 10-μL loop on three selective media, i.e., chromID ESBL (bioMérieux, France), chromID VRE (bioMérieux, France), and chromID CarbaSMART agar (bioMérieux, France), and incubated aerobically at 37°C for 24 h or up to 48 h if no bacterial growth was observed at 24 h. Most of the selective media were loaded with broad-spectrum third-generation cephalosporins (C3). Organisms growing in such conditions are C3 resistant. Organisms were identified by matrix-assisted laser desorption ionization time-of-flight mass spectrometry ([MALDI-TOF MS]; Bruker Biotyper, Bruker Daltonics, Germany). Antimicrobial susceptibility testing was performed on the VITEK 2 platform for *Enterobacteriaceae* (AST-N366 card) and *Enterococcus spp*. (AST-P586 card) and on Mueller-Hinton agar plates by disk diffusion for non-fermenting gram-negative bacteria. The breakpoints recommended by the European Committee on Antimicrobial Susceptibility Testing (EUCAST) were used to assign the following clinical susceptibility categories: susceptible, intermediate, or resistant. Phenotypic confirmation of ESBL production was performed using the combination disk test recommended by the EUCAST. Phenotypic AmpC confirmation tests were performed with the inhibition of AmpC by cloxacillin with Cefotaxime+Cloxacillin Rosco tablets. Carbapenemase production was confirmed by the immunochromatographic lateral flow test RESIST-3 O.K.N. (Coris BioConcept, Belgium).

### Statistical Analysis

Categorical data were summarized as frequency and percentage. For each treatment, changes in the resistance of bacterial strains between visits were compared by McNemar's Chi–squared test. Differences in the proportion of colonization between the treatment groups were analyzed by Chi–squared test for independent variables. A value of *p* < 0.05 was considered statistically significant. All analyses were performed in R version 3.2.0 (R Core Team, 2019).

### Ethical Approval

The protocol of the study was accepted by the Comité d'éthique Hospitalo-Facultaire Universitaire de Liège 707, number 2017-131, which has the legal power to fully agree with the content of the protocol from June 07, 2017. Probiotics are not considered a medicinal product according to European and local guidelines, and this study has no therapeutic claim requiring registration in a database. All patients participating in the trial signed an informed consent form. The study was conducted according to the ICH GCP guidelines and the Declaration of Helsinki. For individuals who were cognitively impaired and/or unable to give informed consent, consent was granted by the legal representative.

## Results

### Study Population

Between September 2017 and October 2019, 703 patients were assessed for eligibility. Out of them, 120 were randomized into one of the three study groups. The follow-up was completed in November 2019 (Figure 1). 75% of the subjects were ≥71 years old and had multiple comorbidities (Table 1). The patients were initially treated by the intravenous route, except for three patients: one in the placebo arm and two in the probiotic mixture arm. The antibiotic treatment was adapted with the adjunction of or switch to another antibiotic in 50 patients after examining their bacterial culture and reading the antibiogram (Table 2). There was no difference in the proportion of intake of other antibiotics among the treatment groups (*p* = 0.429). No significant difference in colonization with C3-resistant bacteria was observed between groups at the time of inclusion (Table 3).

**Table 1:** Patient characteristics.

**Baseline characteristics**	**Placebo *n* = 40**	***Saccharomyces n* = 40**	**Probiotic mixture *n* = 40**
Age average (range)	79.8 (53–97)	76.3 (35–97)	77.3 (48–96)
Gender (male/female)	17/23	16/24	19/21
In hospital stay (days)			
For this infection	11 ± 9	15 ± 11	11 ± 7
For the 10 last years	28 ± 21	33 ± 26	24 ± 16
Comorbidities			
Cancer	13	10	14
Diabetes	9	9	12
Kidney failure	5	7	9
COPD	7	3	3
Smoker (PY ± SD)	8 (27 ± 16)	9 (20 ± 13)	12 (22 ± 14)
Arteritis	15	10	17
Auto-Immunity	3	5	3
Dementia (MMS ± SD)	11 (21 ± 8)	16 (22 ± 7)	19 (24 ± 9)
Katz scale ± SD	16 ± 4	15 ± 6	14 ± 8
Nutritional status			
BMI ± SD	25 ± 7	27 ± 6	26 ± 7
Sarcopenia	14	11	14
Infection origin			
Respiratory	25	23	19
Erysipelas and cellulitis	6	9	8
Urinary tract	6	3	6
Hepato-biliary	3	4	7
Microbiology/colonization			
Positive blood culture	2	3	3
MDR infection	2	0	3
MRSA colonization	3	4	5
Antibiotic treatment			
A-C duration (days)			
IV	3.8 ± 2.1	4.1 ± 2.0	3.5 ± 2.0
Per OS	5.1 ± 8.1	6.1 ± 11.4	4.3 ± 3.4
Adjunction of other antibiotic	15	20	17

*COPD, chronic obstructive pulmonary disease; PY, pack-years; MMS, Mini Mental State; BMI, body mass index; A-C, Amoxicillin-Clavulanate; IV, intravenous route; Per OS, administration by oral route; MDR, Multiple drug resistance; MRSA, Methicillin-resistant Staphylococcus aureus*.

**Table 2:** Adjunction of or switch to other antibiotic treatments.

	**Placebo**	***Saccharomyces***	**Probiotic mixture**
*IV*			
Amoxicillin			1
Flucloxacillin		1	2
Cefazoline		1	1
Cefuroxime	1	3	2
Ceftriaxone	3	2	1
Ceftazidime		2	
Piperacillin-Tazobactam	1	2	1
Temocilin	1	1	
Meropenem			1
Clarithromycin		1	
*Per os*			
Amoxicillin	4	1	1
Flucloxacillin	1		
Cefazoline	1		
Clarithromycin	3	7	2
Clindamycin			1
Doxycycline	2	3	2
INH + rifampicin + myambutol			1
Ciprofloxacin	2	3	2
Metronidazole		1	1
Fluconazole	2		

*A-C, Amoxicillin-Clavulanate; IV, intravenous route; Per OS, administration by oral route; INH, Isoniazid. There are no statistical differences between the groups*.

**Table 3:** *Post-hoc* analysis of bacterial cultures.

**Patient code**	**Culture**	**Site of collection**
**Placebo**		
8	*E coli* ESBL*, K pneumoniae*	Urine
26	*Unidentified*	Respiratory
31	*E coli* ESBL	Urine
34	*Acinetobacter*	Scab
63	*Unidentified*	Respiratory
69	*M morganii, P stuartii, P aeruginosa*	Urine
89	*E coli, K pneumoniae*	Urine, scab
108	*K pneumoniae, E coli* ESBL	Urine
112	*P aeruginosa*	Sputum
120	*K pneumoniae, P aeruginosa*	Urine
***Saccharomyces***	
7	*E coli* ESBL	Blood culture
9	*P aeruginosa*	Urine
15	*M morganii*	Repiratory
18	*P aeruginosa*	Blood culture
66	*E coli*	Urine
67	*E coli*	Urine
76	*E coli, P aeruginosa*	Urine
104	*E coli*	Urine
105	*E cloacae*	Urine
107	*E coli, E cloacae*	Soft tissue abscess
**Probiotic mixture**	
16	*E coli*	Urine
25	*E coli*	Urine
28	*Aerococcus*	Urine
61	*E colacae*	Urine
68	*C freundii*	Urine
75	*E coli, M morganii*	Urine, scab
84	*E coli*	Urine
95	*P aeruginosa*	Sputum
124	*Klebsiella*	Urine
125	*P aeruginosa*	Sputum

*Bacterial culture obtained from infectious sites after the end of the trial period*.

Six months after the inclusion of the last patient in the trial, we reviewed the bacterial cultures for all the included patients using samples isolated from infectious sources. This represents a follow up period of up to 2 years and 10 positive cultures in each arm. In the placebo arm, ESBL-producing bacteria were retrieved in three urinary cultures, and *Pseudomonas aeruginosa* was retrieved in two urinary cultures and one sputum culture. In the *Saccharomyces* arm, ESBL-producing bacteria were retrieved in one blood culture, and *Pseudomonas aeruginosa*, in two urine cultures and one blood culture. However, in the probiotic mixture arm, ESBL-producing bacteria were not retrieved in any of the cultures. *Pseudomonas aeruginosa* was retrieved in two sputum cultures (Table 3). This observation is purely informative as the trial was not designed to identify prevention of infection with multi-drug resistant bacteria.

The survival rate was comparable in all treatment groups (*p* = 0.460).

### Outcome of the Intervention

#### Colonization With C3-Resistant Non-fermenting Bacteria

In the placebo arm, 28.9% (11/38) of patients were colonized with non-fermenting bacteria at visit 2 compared with 23.7% (9/38) at visit 1 (n.s.). In the *Saccharomyces* arm, 22.2% (8/36) of the patients were colonized at visit 2 compared to 33.3% (12/36) at visit 1 (n.s.).

In the probiotic mixture arm, 8.3% (3/36) of the patients were colonized at visit 2 as compared to 25% (9/36) at visit 1, showing a significant decrease in colonization (*p* = 0.041). This phenomenon was associated with specific decolonization of *Pseudomonas* (visit 1 vs. visit 2, *p* = 0.041). No new colonization with *Pseudomonas* was observed from visit 1 to visit 2. This trend was observed throughout the whole study duration as 18.5% (5/27) of the patients were colonized at visit 3 (visit 2 vs. visit 3, *p* = 0.617) compared to 30% (8/26) in the placebo arm and 36% (9/25) in the *Saccharomyces* arm.

#### Colonization With C3-Resistant Enterobacteria

In the placebo and *Saccharomyces* arms, the colonization rate with C3-resistant enterobacteria between visit 1 and visit 2 increased, respectively, from 15.8% (6/38) and 22.2% (8/36) to 29% (11/38) and 33.3% (12/36) (*p* = n.s.) (Table 4). No significant change in the colonization rate was observed at the end of the trial.

**Table 4:** C3-resistant bacteria.

	**Placebo**			***Saccharomyces***			**Probiotic mixture**		
	***V1 n = 40***	***V2 n = 38***	***V3 n = 27***	***V1 n = 40***	***V2 n = 36***	***V3 n = 25***	***V1 n = 40***	***V2 n = 36***	***V3 n = 27***
***ESBL producing bacteria***									
*E. coli*	2	2	2	2	4	2	7	7	5
*Klebsiella pneumoniae*	2	3					2	1	1
*Morganella morganii*	1	1	1						
*Citrobacter freundii*				1				1	
*Citrobacter farmeri*								1	
*Enterobacter cloacae*					2				
***AmpC producing bacteria***									
*E. coli*		1	1			2	1	3	
*Morganella morganii*		1		1			1	2	
*Hafnia alvei*		1						1	1
*Enterobacter aerogenes*		1	3						
*Enterobacter cloacae*				1	3	1	1	4	
*Citrobacter freundii*	1	1		2	2	2		1	
*Citrobacter brakii*				1	1	1		1	
*Klebsiella aerogenes*					1	1			
***Non-fermenting bacteria***									
*Pseudomonas aeruginosa*	7	8	8	9	5	5	11	2	4
*Pseudomonas spp*.	2		1	2	2	2	1	1	
*Pseudomonas putida*		1	2			1			1
*Pseudomonas citronellolis*						1			
*Stenotrophomonas maltophila*		1	2	1	1	2			
*Acinetobacter pitii*		2							2
*Achromobacter spp*.		1	1						
*Ochrobatrum intermedium*						1			

*Identification of the bacterial stains in stool cultures. In some cultures, multiple stains and mechanisms of resistance were retrieved. Six cultures of non-fermenting bacteria demonstrated resistance for narrow-spectrum C3 directed against Pseudomonas and carbapenems: in the placebo arm 2 at visit 1 and 1 at visit 3, in the Saccharomyces arm 1 at visit 1 and 1 at visit 3 and in the probiotic mixture 1 at visit 3*.

**Figure 2:**
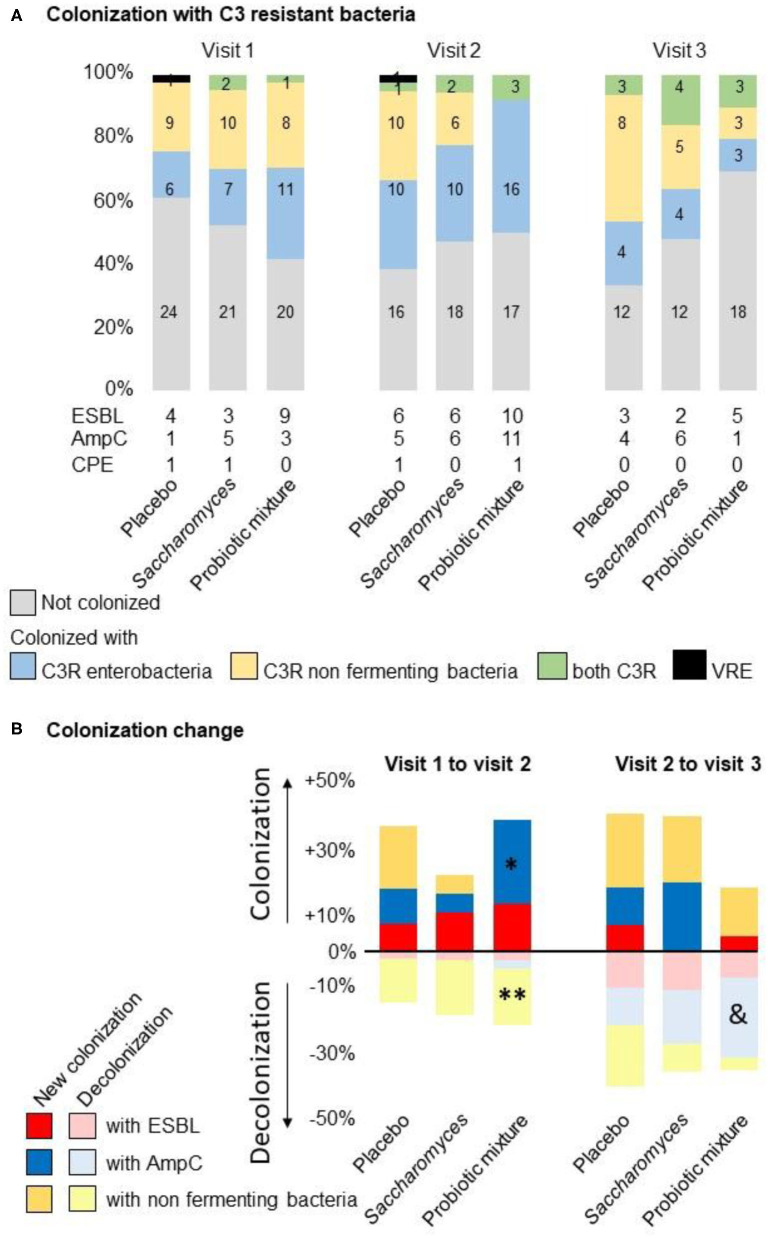
Colonization by treatment group. **(A)** Colonization with broad spectrum C3-resistant bacteria. In all groups of treatment, the proportion of patients colonized with C3 resistant enterobacteria increase at visit 2. In the Probiotic mixture group, this increase was more important due to a population of enterobacteria showing the phenotypic characteristics of expression of AmpC. The colonization with *Pseudomonas* decreased strongly in the Probiotic mixture group at visit 2. Colonization with C3 resistant bacteria returned to the basal levels at visit 3. **(B)** Changes in the rate of colonization from visit 1 to 2 or visit 2 to 3: (*) increased the proportion of C3-resistant bacteria in the probiotic mixture arm between visit 1 and visit 2, confirmed by a 25% increase in AmpC-producing enterobacteria (*p* = 0.027). (**) Paired analysis of colonization with C3-resistant bacteria shows a 16% reduction of colonization with non-fermenting bacteria in the probiotic mixture arm between visit 1 and visit 2, explained by a reduction in *Pseudomonas* species (*p* = 0.041). (&) Reduction in colonization by 23.1% after the study treatment was observed at visit 3 in the probiotic mix arm, corresponding to the proportion of patients who were colonized at visit 1 (*p* = 0.041).

In the probiotic mixture arm, the colonization rate increased from 25% (9/36) to 52.7% (19/36) (*p* = 0.016). This increase in the resistance is explained by a 22.9% increase in colonization with AmpC-producing enterobacteria (visit 2 vs. visit 1, *p* = 0.027) in the probiotic mixture arm, as compared to a 10.2% increase in the placebo arm and a 5.4% increase in the *Saccharomyces* arm (n.s.). The proportion of patients colonized with AmpC-producing enterobacteria were stable between visit 2 and visit 3 in the placebo and *Saccharomyces* arms (n.s.) while a significant decline was seen in the probiotic mixture arm to 3.8% at visit 3 (*p* = 0.041) (Figure 2).

#### Colonization With ESBL- or Carbapenemase-Producing Bacteria and VRE

The prevalence of colonization with ESBL-producing enterobacteria increased at the end of the antibiotic treatment in the placebo, *Saccharomyces* and probiotic mixture arms from 10.3% (4/39), 7.7% (3/39), and 23.1% (9/39) to 15.4% (6/39), 16.7% (6/36), and 27.8% (10/36), respectively (n.s.). The colonization rates were normalized to the initial values at visit 3 (11.1% [3/27], 8.0% [2/25], and 19.2% [5/26], respectively).

Only one patient was colonized with CPE-producing bacteria in the probiotic mixture arm after antibiotic therapy, but showed negative results after the study treatment. One patient in the placebo and one in the *Saccharomyces* arm were already colonized with CPE-producing bacteria at the time of inclusion, and both showed negative results at visit 3 and visit 2, respectively (Table 4). One patient was colonized with VRE at the time of inclusion in the placebo arm that VRE was not retrieved at visit 3.

## Discussion

Most of the patients were elderly and had important comorbidities. Stool collection was impossible at home for these patients, explaining the lack of follow-up between visits 2 and 3. Blackcurrant leaf and fruit in the composition of the probiotic mixture were not taken into account for the preparation of the placebo. As a prebiotic, its effects on *Pseudomonas* are limited to phenotypic changes as biofilm formation. Considering the low concentration in the preparation, its effect on the microbiome is negligible (16).

We observed an overrepresentation of enterobacteria with the phenotypic characteristic of AmpC expression in the stool sample collected after the antibiotic treatment (visit 2) in the probiotic mixture group. AmpC is known to be upregulated in enterobacteria in the presence of amoxicillin-clavulanate and could therefore be due near the end of the antibiotic therapy, (17, 18) AmpC should be considered as an enzyme implicated in the homeostasis of the bacterial cell wall. Its expression depends on the concentration in cell wall fragments in the cytosol. When the degradation of the cell wall accelerates, the expression of AmpC increases in a parallel manner for muropeptides or peptidoglycans. As *Lactobacillus* and *Bifidobacterium* are susceptible to amoxicillin-clavulanate, their actions on enterobacteria could be mediated by muropeptides and peptidoglycans released after the death of the probiotic bacteria which could be linked to the probiotic mixture group (19). None of the patients showed infection with AmpC-producing bacteria.

In this study, a reduction in colonization with *Pseudomonas* was seen throughout the duration of probiotic mixture treatment. Other studies have also confirmed this trend in wound and respiratory tract infections in patients treated with *L. plantarum* and *L. rhamnosus*, respectively (20, 21). Probiotic bacteria may directly affect the proliferation of MDR by producing antimicrobial compounds such as short-chain fatty acids, hydrogen peroxide, nitric oxide, or bacteriocin or indirectly affect it by modifying the intestinal epithelial barrier, by adherence, or by competing for the substrate (22, 23). The predominance of *Lactobacillus spp*. in the gut microbiota was also observed in patients who did not acquire VRE after in-hospital antibiotic treatment (24).

Therefore, it seems that probiotic treatment should not be provided as a single method of treatment, but should involve specific treatments depending on the probiotic bacteria, with adapted posology. Probiotic mixtures composed of lactobacilli and bifidobacteria should be given before and after the antibiotic treatment to prevent colonization with bacterial populations resistant to the antibiotics.

## Summary of the Article's Main Point

Treatment with a probiotic mixture associating *lactobacilli, bifidobacteria* and *Saccharomyces* during an in-hospital antibiotic treatment is associated with a decrease in colonization of the gut microbiota with *Pseudomonas* and with a transient colonization with AmpC producing enterobacteria after the antibiotic treatment which was not retrieved at the end of the study treatment.

## Data Availability Statement

The raw data supporting the conclusions of this article will be made available by the authors, without undue reservation.

## Ethics Statement

The protocol of the study was accepted by the Comité d'éthique Hospitalo-Facultaire Universitaire de Liège 707, number 2017-131 that has legal power to fully agreed with the content of the protocol on the 7th of June 2017. Probiotics are not considered as medicinal product in accordance with European and local guidelines and this study has no therapeutic claim. As such, this research is not considered a clinical trial, and registration in an official database is not required. All patients participating in the trial signed an informed consent form. The study was conducted according to the ICH GCP guidelines and the Declaration of Helsinki. For individuals who were cognitively impaired and/or unable to give informed consent, consent was granted by the legal representative.

## Author Contributions

GW: conception and design of the study, acquisition, analysis, and interpretation of data, drafting the article, revising the article critically for intellectual content, and guarantor of the data. VV: analysis and interpretation of data. MVDD: conception and design of the study, analysis, and interpretation of data. EM: analysis and interpretation of data. GV: analysis and interpretation of data. J-CM: critical revision of the article for intellectual content. PDC: conception and design of the study and critical revision of the article for intellectual content.

## Conflict of Interest

GW reports grants from Metagenics, during the conduct of the study. MV and GV reports non-financial support from Metagenics Europe, during the conduct of the study and an employee of Metagenics Europe, distributor of Bactiol duo used in this trial. PC is inventor on patent applications dealing with the use of A.muciniphila and its components in the treatment of obesity and related disorders and co-founder of A-Mansia biotech SA. The remaining authors declare that the research was conducted in the absence of any commercial or financial relationships that could be construed as a potential conflict of interest.
