# Supplementary material for: Do Probiotics During In-Hospital Antibiotic Treatment Prevent Colonization of Gut Microbiota With Multi-Drug-Resistant Bacteria? A Randomized Placebo-Controlled Trial Comparing Saccharomyces to a Mixture of Lactobacillus, Bifidobacterium, and Saccharomyces
Source: Front Public Health. 2021 Mar 8;8:578089. doi: 10.3389/fpubh.2020.578089 (PMC7982943; doi:10.3389/fpubh.2020.578089)
Supplement: Supplementary file 1 [file Data_Sheet_1.PDF]

Annex 1: Clinical trial Application Form

REQUEST FOR AUTHORISATION OF A CLINICAL TRIAL ON A MEDICINAL PRODUCT FOR HUMAN USE TO THE COMPETENT AUTHORITIES AND FOR OPINION OF THE ETHICS COMMITTEES IN THE COMMUNITY

To be filled in by the applicant
The questions in this form for the request for authorisation from the Competent Authority are also relevant for the opinion from an Ethics Committee (it represents module 1 of the form for applying to an ethics committee) and can be used as part of that application. Please indicate the relevant purpose in a box below.

REQUEST FOR AUTHORISATION TO THE COMPETENT AUTHORITY: **Yes •**
REQUEST FOR OPINION OF THE ETHICS COMMITTEE: **No •**

A. TRIAL IDENTIFICATION

A.1	Member State in which the submission is being made:		**Belgium - FPS Health-DGM**
A.2	EudraCT number:		**2017-001814-27**
A.3	Full title of the trial:		
	English	**Prevention of side effects caused by an antibiotic treatment during an hospitalisation via the concomitant administration of probiotics.**	
	French	**Prévention d'effets non désirés causés par une antibiothérapie durant une hospitalisation par l'administration concomitante de probiotiques.**	
A.3.1	Title of the trial for lay people, in easily understood, i.e. non-technical, language:		
	English	**Does probiotics decrease the risk of being colonised of antibiotic resistant bacteria in the gut flora, and the digestive discomfort caused by an antibiotic treatment during a stay in the hospital?**	
	French	**Un traitement par des probiotiques diminue t'il le risque d'être colonisé par des bactéries résistantes aux antibiotiques dans la flore du colon et l'inconfort digestif causé par un traitement antibiotique durant un séjour à l'hôpital?**	
A.3.2	Name or abbreviated title of the trial where available:		
	English	**Prevention with probiotics during an antibiotic treatment in the hospital.**	
	French	**Prévention par des probiotiques durant un traitement antibiotique à l'hôpital.**	
A.4	Sponsor's protocol code number, version and date[1]:		
A.4.1	Sponsor's protocol code number:		**01.15**
A.4.2	Sponsor's protocol version:		
A.4.3	Sponsor's protocol date:		**2017-04-28**
A.5	Additional international study identifiers (e.g. WHO, ISRCTN[2] , US NCT Number[3]) if available		
A.5.1	ISRCTN number:		
A.5.2	US NCT number:		
A.5.3	WHO Universal Trial Number (UTN):		
A.5.4	Other Identifier:		
A.6	Is this a resubmission?		**No •**
	If 'Yes', indicate the resubmission letter[4]:		**First Submission**
A.7	Is the trial part of an agreed Paediatric Investigation Plan?		**No •**
A.8	EMA Decision number of Paediatric Investigation Plan:		

B. IDENTIFICATION OF THE SPONSOR RESPONSIBLE FOR THE REQUEST

**B.1**	**SPONSOR**	
B.1.1	Name of organisation:	**Metagenics**
B.1.2	Name of the person to contact:	
B.1.2.1	Given name	**Mieke**
B.1.2.2	Middle name	
B.1.2.3	Family name	**Van Den Driessche**
B.1.3	Address:	
B.1.3.1	Street address	**Edward Vlietinckstraat, 20**
B.1.3.2	Town/city	**Oostende**
B.1.3.3	Post code	
B.1.3.4	Country	**Belgium**
B.1.4	Telephone number:	**0032 59 805824**
B.1.5	Fax number:	**0032 59 807083**
B.1.6	E-mail:	

**B.2**	**LEGAL REPRESENTATIVE[5] OF THE SPONSOR IN THE COMMUNITY FOR THE PURPOSE OF THIS TRIAL** (if different from the sponsor)
B.2.1	Name of organisation:
B.2.2	Name of person to contact:
B.2.2.1	Given name
B.2.2.2	Middle name
B.2.2.3	Family name
B.2.3	Address:
B.2.3.1	Street address
B.2.3.2	Town/city
B.2.3.3	Post code
B.2.3.4	Country
B.2.4	Telephone number:
B.2.5	Fax number:
B.2.6	E-mail:

**B.3**	**STATUS OF THE SPONSOR:**	
B.3.1	Commercial:	**Yes •**
B.3.2	Non commercial:	**No •**

**B.4**	**Source(s) of Monetary or Material Support for the clinical trial (repeat as necessary):**	
B.4.1	Name of organisation:	**Metagenics**
B.4.2	Country:	**Belgium**

**B.5**	**Contact point[6] designated by the sponsor for further information on the trial**	
B.5.1	Name of organisation:	**Clinique Saint Pierre Ottignies**
B.5.2	Functional name of contact point (e.g. "Clinical Trial Information Desk"):	**Principal Investigator**
B.5.3	Address:	
B.5.3.1	Street address	**Avenue reine Fabiola, 9**
B.5.3.2	Town/city	**Ottignies**
B.5.3.3	Post code	
B.5.3.4	Country	**Belgium**
B.5.4	Telephone number:	**0032 10 437493**
B.5.5	Fax number:	
B.5.6	E-mail: (use a functional e-mail address rather than a personal one)	**gregoire.wieers@cspo.be**

C. APPLICANT IDENTIFICATION, (please tick the appropriate box)

**C.1**	**REQUEST FOR THE COMPETENT AUTHORITY**		
C.1.1	Sponsor		
C.1.2	Legal representative of the sponsor		
C.1.3	Person or organisation authorised by the sponsor to make the application		**Yes •**
C.1.4	Complete the details of the applicant below even if they are provided elsewhere on the form:		
C.1.4.1	Name of Organisation:	**Clinique Saint Pierre Ottignies**	
C.1.4.2	Name of contact person:		
C.1.4.2.1	Given name	**Grégoire**	
C.1.4.2.2	Middle name	**Jacques François Ghislain**	
C.1.4.2.3	Family name	**Wieërs**	
C.1.4.3	Address:		
C.1.4.3.1	Street address	**Avenue Reine Fabiola, 9**	
C.1.4.3.2	Town/city	**Ottignies**	
C.1.4.3.3	Post code	**1340**	
C.1.4.3.4	Country	**Belgium**	
C.1.4.4	Telephone number:	**0032 10 437493**	
C.1.4.5	Fax number:		
C.1.4.6	E-mail:	**gregoire.wieers@cspo.be**	
C.1.5	Request to receive a copy of CTA data as XML:		
C.1.5.1	Do you want a copy of the CTA form data saved on EudraCT as an XML file?		**No •**
C.1.5.1.1	If Yes provide the e-mail address(es) to which it should be sent (up to 5 addresses):		
C.1.5.1.2	Do you want to receive this via password protected link(s)[7]?		**No •**
If you answer No to question C.1.5.1.2 the .xml file will be transmitted by less secure e-mail link(s)			

D. INFORMATION ON EACH IMP

Information on each 'bulk product' before trial-specific operations (blinding, trial specific packaging and labelling) should be provided in this section for each investigational medicinal product (IMP) being tested including each comparator and each placebo, if applicable. ***For placebo go directly to D.8****. If the trial is performed with several products use extra pages and give each product a sequential number in D.1.1. If the product is a combination product, information should be given for each active substance.*

**D.1**	**IMP IDENTIFICATION**	
Indicate which of the following is described below, then repeat as necessary for each of the numbered IMPs to be used in the trial (assign numbers from 1-n):		
D.1.1	This refers to the IMP number:	**PR1**
D.1.2	IMP being tested	**Yes •**
D.1.3	IMP used as a comparator	**No •**

**D.2**	**STATUS OF THE IMP**	
D.2.1	Has the IMP to be used in the trial a marketing authorisation?	**Yes •**
**If the IMP has a marketing authorisation in the Member State concerned by this application, but the trade name and marketing authorisation holder are not fixed in the protocol, go to section D.2.2.**		
D.2.1.1	If 'Yes', specify the product to be used in the clinical trial:	
D.2.1.1.1	Trade name	**Probactiol Duo**
D.2.1.1.1.1	EV Product Code (where applicable)	
D.2.1.1.2	Name of the Marketing Authorisation Holder:	**Metagenics**
D.2.1.1.3	Marketing Authorisation number (if Marketing Authorisation granted by a Member State):	
D.2.1.1.4	Is the IMP modified in relation to its Marketing Authorisation?	**No •**
D.2.1.1.4.1	If 'Yes', please specify:	
D.2.1.2	The country that granted the Marketing Authorisation	**Belgium**
D.2.1.2.1	Is this the Member State concerned with this application?	**Yes •**

D.2.2	Situations where an IMP to be used in the CT has a Marketing Authorisation in the Member State concerned, but the protocol allows that any brand of the IMP with a Marketing Authorisation in that Member State be administered to the trial subjects and it is not possible to clearly identify the IMP(s) in advance of the trial start	
D.2.2.1	In the protocol, is treatment defined only by active substance?	**No •**
D.2.2.1.1	If 'Yes', give active substance in D.3.8 or D.3.9	
D.2.2.2	In the protocol, do treatment regimens allow different combinations of marketed products used according to local clinical practice at some or all investigator sites in the MS?	**No •**
D.2.2.2.1	If 'Yes', give active substance in D.3.8 or D.3.9	
D.2.2.3	The products to be administered as IMPs are defined as belonging to an ATC group[9]	**No •**
D.2.2.3.1	If 'Yes', give the ATC group of the applicable authorised codes in the ATC code field (level 3 or the level that can be defined) in D.3.3	
D.2.2.4	Other:	**Yes •**
D.2.2.4.1	If 'Yes', please specify: **Probactiol duo is a food supplement composed with Saccharomyces boulardii 6x10^9 CFU/dose, Lactobacillus acidophilus NCFM 2x10^9 CFU/dose, Bifidobacterium animalis subsp. lactis Bi-07 2x10^9 CFU/dose, Bifidobacterium animalis subsp. lactis Bi-04 2x10^9 CFU/dose, Lactobacillus paracasei Lpc-37 2x10^9 CFU/dose, blackberry fruit and leaf 50 mg, vitamin D (cholecalciferol) 1.25 µg, excipients microcrystalline cellulose 40 mg, silicon dioxide 4 mg, magnesium stearate 4 mg.**	

D.2.3	IMPD submitted:	
D.2.3.1	Full IMPD:	**No •**
D.2.3.2	Simplified IMPD:	**No •**
D.2.3.3	Summary of product characteristics (SmPC) only:	**Yes •**
D.2.4	Has the use of the IMP been previously authorised in a clinical trial conducted by the sponsor in the Community?	**Not Answered •**
D.2.4.1	If 'Yes' specify which Member States:	
D.2.5	Has the IMP been designated in this indication as an orphan drug in the Community?	**No •**
D.2.5.1	If 'Yes', give the orphan drug designation number[10]**:**	

D.2.6	Has the IMP been the subject of scientific advice related to this clinical trial?	**No •**
D.2.6.1	If 'Yes' to D.2.6, please indicate source of advice and provide a copy in the CTA request:	
D.2.6.1.1	CHMP[11]?	**No •**
D.2.6.1.2	National Competent Authority?	**No •**

**D.3**	**DESCRIPTION OF THE IMP**	
D.3.1	Product name where applicable[12]:	
D.3.2	Product code where applicable[13]:	
D.3.3	ATC codes, if officially registered[14]:	
D.3.4	Pharmaceutical form (use standard terms):	**Capsule**
D.3.4.1	Is this a specific paediatric formulation?	**No •**
D.3.5	Maximum duration of treatment of a subject according to the protocol: **30 days**	
D.3.6	Dose allowed:	
D.3.6.1	For first trial only:	
	Specify per day or total	**Not Answered •**
	Specify total dose (number and unit):	
	Route of administration (relevant to the first dose):	
D.3.6.2	For all trials	
	Specify per day or total	**Per day •**
	Specify total dose (number and unit):	**28.10^6 thousand CFU thousand colony forming units**
	Route of administration (relevant to the maximum dose):	**Oral use**
D.3.7	Routes of administration (use standard terms):	**Oral use**

D.3.8	Name of each active substance (INN or proposed INN if available):
D.3.9	Other available name for each active substance (provide all available):
D.3.9.1	CAS[15] number
D.3.9.2	Current sponsor code
D.3.9.3	Other descriptive name
D.3.9.4	EV Substance code
D.3.9.5	Full Molecular formula
D.3.9.6	Chemical/biological description of the Active Substance
D.3.10	Strength (specify all strengths to be used):
D.3.10.1	Concentration unit:
D.3.10.2	Concentration type ("exact number", "range", "more than" or "up to"):
D.3.10.3	Concentration (number).

D.3.11	Type of IMP	
Does the IMP contain an active substance:		
D.3.11.1	Of chemical origin?	**No •**
D.3.11.2	Of biological / biotechnological origin (other than	**Yes •**

	Advanced Therapy IMP (ATIMP)?	
Is this a:		
D.3.11.3	Advanced Therapy IMP (ATIMP)?	**No •**
D.3.11.3.1	Somatic cell therapy medicinal product[16]?	**No •**
D.3.11.3.2	Gene therapy medicinal product[17]?	**No •**
D.3.11.3.3	Tissue Engineered Product[18]?	**No •**
D.3.11.3.4	Combination ATIMP (i.e. one involving a medical device[19])?	**No •**
D.3.11.3.5	Has the Committee on Advanced Therapies issued a classification for this product?	**No •**
D.3.11.3.5.1	If 'Yes' please provide that classification and its reference number:	
D.3.11.4	Combination product that includes a device, but does not involve an Advanced Therapy?	**No •**
D.3.11.5	Radiopharmaceutical medicinal product?	**No •**
D.3.11.6	Immunological medicinal product (such as vaccine, allergen, immune serum)?	**No •**
D.3.11.7	Plasma derived medicinal product?	**No •**
D.3.11.8	Extractive medicinal product?	**No •**
D.3.11.9	Recombinant medicinal product?	**No •**
D.3.11.10	Medicinal product containing genetically modified organisms?	**No •**
D.3.11.10.1	Has the authorisation for contained use or release been granted?	**No •**
D.3.11.10.2	Is it pending?	**No •**
D.3.11.11	Herbal medicinal product?	**Yes •**
D.3.11.12	Homeopathic medicinal product?	**No •**
D.3.11.13	Another type of medicinal product?	**Yes •**
D.3.11.13.1	If 'another type of medicinal product' specify the type of medicinal product: **Mixture of probiotic bacteria and yeast**	
D.3.12	Mode of action (*free text*[20]) **Probiotic bacteria and yeast influence the commensal bacteria in the gut.**	
D.3.13	Is it an IMP to be used in a first-in-human clinical trial?	**No •**
D.3.13.1	If 'Yes', are there risk factors identified, according to the guidance FIH?[21]	

**D.4**	**SOMATIC CELL THERAPY INVESTIGATIONAL MEDICINAL PRODUCT (NO GENETIC MODIFICATION)**	
D.4.1	Origin of cells	
D.4.1.1	Autologous	**No •**
D.4.1.2	Allogeneic	**No •**
D.4.1.3	Xenogeneic	**No •**
D.4.1.3.1	If 'Yes', specify the species of origin:	
D.4.2	Type of cells	
D.4.2.1	Stem cells	**No •**
D.4.2.2	Differentiated cells	**No •**
D.4.2.2.1	If 'Yes', specify the type (e.g. keratinocytes, fibroblasts, chondrocytes…):	
D.4.2.3	Others:	**No •**
D.4.2.3.1	If others, specify:	

**D.5**	**GENE THERAPY INVESTIGATIONAL MEDICINAL PRODUCTS**	
D.5.1	Gene(s) of interest:	
D.5.2	In vivo gene therapy:	**No •**
D.5.3	Ex vivo gene therapy:	**No •**
D.5.4	Type of gene transfer product	
D.5.4.1	Nucleic acid (e.g. plasmid): If 'Yes', specify if:	**No •**

D.5.4.1.1	Naked:	**No •**
D.5.4.1.2	Complexed	**No •**
D.5.4.2	Viral vector:	**No •**
D.5.4.2.1	If 'Yes', specify the type: adenovirus, retrovirus, AAV, …:	
D.5.4.3	Others	**No •**
D.5.4.3.1	If others, specify:	
D.5.5	Genetically modified somatic cells:	**No •**
If 'Yes', specify the origin of the cells:		
D.5.5.1	Autologous:	**No •**
D.5.5.2	Allogeneic:	**No •**
D.5.5.3	Xenogeneic:	**No •**
D.5.5.3.1	If 'Yes', specify the species of origin:	
D.5.5.4	Specify type of cells (hematopoietic stem cells…):	

**D.6**	**TISSUE ENGINEERED PRODUCT**	
The indication which determines that this is a Tissue Engineered Product as opposed to a Cell Therapy product is given in section E.1.1.		
D.6.1	Origin of cells	
D.6.1.1	Autologous	**No •**
D.6.1.2	Allogeneic	**No •**
D.6.1.3	Xenogeneic	**No •**
D.6.1.3.1	If 'Yes', specify the species of origin:	
D.6.2	Type of cells	
D.6.2.1	Stem cells	**No •**
D.6.2.2	Differentiated cells	**No •**
D.6.2.2.1	If 'Yes', specify the type of cells(e.g. keratinocytes, fibroblasts, chondrocytes, …):	
D.6.2.3	Others:	**No •**
D.6.2.3.1	If others, specify:	

**D.7**	**PRODUCTS CONTAINING DEVICES (i.e. MEDICAL DEVICES, SCAFFOLDS ETC.)**	
D.7.1	Give a brief description of the device:	
D.7.2	What is the name of the device?	
D.7.3	Is the device implantable?	**No •**
D.7.4	Does this product contain:	
D.7.4.1	A medical device?	**No •**
D.7.4.1.1	Does this medical device have a CE mark?	**No •**
D.7.4.1.1.1	The notified body is:	
D.7.4.2	Bio-materials?	**No •**
D.7.4.3	Scaffolds?	**No •**
D.7.4.4	Matrices?	**No •**
D.7.4.5	Other?	**No •**
D.7.4.5.1	If other, specify:	

**D.1**	**IMP IDENTIFICATION**	
Indicate which of the following is described below, then repeat as necessary for each of the numbered IMPs to be used in the trial (assign numbers from 1-n):		
D.1.1	This refers to the IMP number:	**PR2**
D.1.2	IMP being tested	**No •**
D.1.3	IMP used as a comparator	**Yes •**

D.2	STATUS OF THE IMP	
D.2.1	Has the IMP to be used in the trial a marketing authorisation?	**Yes •**

If the IMP has a marketing authorisation in the Member State concerned by this application, but the trade name and marketing authorisation holder are not fixed in the protocol, go to section D.2.2.

D.2.1.1	If 'Yes', specify the product to be used in the clinical trial:	
D.2.1.1.1	Trade name	**Enterol**
D.2.1.1.1.1	EV Product Code (where applicable)	
D.2.1.1.2	Name of the Marketing Authorisation Holder:	**Biocodex**
D.2.1.1.3	Marketing Authorisation number (if Marketing Authorisation granted by a Member State):	
D.2.1.1.4	Is the IMP modified in relation to its Marketing Authorisation?	**No •**
D.2.1.1.4.1	If 'Yes', please specify:	
D.2.1.2	The country that granted the Marketing Authorisation	**Belgium**
D.2.1.2.1	Is this the Member State concerned with this application?	**Yes •**

D.2.2	Situations where an IMP to be used in the CT has a Marketing Authorisation in the Member State concerned, but the protocol allows that any brand of the IMP with a Marketing Authorisation in that Member State be administered to the trial subjects and it is not possible to clearly identify the IMP(s) in advance of the trial start	
D.2.2.1	In the protocol, is treatment defined only by active substance?	**No •**
D.2.2.1.1	If 'Yes', give active substance in D.3.8 or D.3.9	
D.2.2.2	In the protocol, do treatment regimens allow different combinations of marketed products used according to local clinical practice at some or all investigator sites in the MS?	**No •**
D.2.2.2.1	If 'Yes', give active substance in D.3.8 or D.3.9	
D.2.2.3	The products to be administered as IMPs are defined as belonging to an ATC group[9]	**No •**
D.2.2.3.1	If 'Yes', give the ATC group of the applicable authorised codes in the ATC code field (level 3 or the level that can be defined) in D.3.3	
D.2.2.4	Other:	**Yes •**
D.2.2.4.1	If 'Yes', please specify: **Enterol is a diet supplement composed with Saccharomyces boulardii 6x10^9 CFU/dose, excipients mannitol 60 mg, magnesium stearate 6.7 mg, silicon dioxide 3 mg.**	

D.2.3	IMPD submitted:	
D.2.3.1	Full IMPD:	**No •**
D.2.3.2	Simplified IMPD:	**No •**
D.2.3.3	Summary of product characteristics (SmPC) only:	**Yes •**
D.2.4	Has the use of the IMP been previously authorised in a clinical trial conducted by the sponsor in the Community?	**Not Answered •**
D.2.4.1	If 'Yes' specify which Member States:	
D.2.5	Has the IMP been designated in this indication as an orphan drug in the Community?	**No •**
D.2.5.1	If 'Yes', give the orphan drug designation number[10]**:**	

D.2.6	Has the IMP been the subject of scientific advice related to this clinical trial?	**No •**
D.2.6.1	If 'Yes' to D.2.6, please indicate source of advice and provide a copy in the CTA request:	
D.2.6.1.1	CHMP[11]?	**No •**
D.2.6.1.2	National Competent Authority?	**No •**

D.3	DESCRIPTION OF THE IMP	
D.3.1	Product name where applicable[12]:	
D.3.2	Product code where applicable[13]:	
D.3.3	ATC codes, if officially registered[14]:	
D.3.4	Pharmaceutical form (use standard terms):	**Capsule**
D.3.4.1	Is this a specific paediatric formulation?	**No •**
D.3.5	Maximum duration of treatment of a subject according to the protocol: **30**	
D.3.6	Dose allowed:	
D.3.6.1	For first trial only:	
	Specify per day or total	**Not Answered •**
	Specify total dose (number and unit):	
	Route of administration (relevant to the first dose):	
D.3.6.2	For all trials	
	Specify per day or total	**Per day •**
	Specify total dose (number and unit):	**12.10^6 million CFU million colony forming units**
	Route of administration (relevant to the maximum dose):	**Oral use**
D.3.7	Routes of administration (use standard terms):	**Oral use**

D.3.8	Name of each active substance (INN or proposed INN if available):
D.3.9	Other available name for each active substance (provide all available):
D.3.9.1	CAS[15] number
D.3.9.2	Current sponsor code
D.3.9.3	Other descriptive name
D.3.9.4	EV Substance code
D.3.9.5	Full Molecular formula
D.3.9.6	Chemical/biological description of the Active Substance
D.3.10	Strength (specify all strengths to be used):
D.3.10.1	Concentration unit:
D.3.10.2	Concentration type ("exact number", "range", "more than" or "up to"):
D.3.10.3	Concentration (number).

D.3.11	Type of IMP	
Does the IMP contain an active substance:		
D.3.11.1	Of chemical origin?	**No •**
D.3.11.2	Of biological / biotechnological origin (other than Advanced Therapy IMP (ATIMP)?	**Yes •**
Is this a:		
D.3.11.3	Advanced Therapy IMP (ATIMP)?	**No •**
D.3.11.3.1	Somatic cell therapy medicinal product[16]?	**No •**
D.3.11.3.2	Gene therapy medicinal product[17]?	**No •**
D.3.11.3.3	Tissue Engineered Product[18]?	**No •**
D.3.11.3.4	Combination ATIMP (i.e. one involving a medical device[19])?	**No •**
D.3.11.3.5	Has the Committee on Advanced Therapies issued a classification for this product?	**No •**
D.3.11.3.5.1	If 'Yes' please provide that classification and its reference number:	
D.3.11.4	Combination product that includes a device, but does not involve an Advanced Therapy?	**No •**
D.3.11.5	Radiopharmaceutical medicinal product?	**No •**
D.3.11.6	Immunological medicinal product (such as vaccine, allergen, immune serum)?	**No •**
D.3.11.7	Plasma derived medicinal product?	**No •**
D.3.11.8	Extractive medicinal product?	**No •**
D.3.11.9	Recombinant medicinal product?	**No •**

D.3.11.10	Medicinal product containing genetically modified organisms?	**No •**
D.3.11.10.1	Has the authorisation for contained use or release been granted?	**No •**
D.3.11.10.2	Is it pending?	**No •**
D.3.11.11	Herbal medicinal product?	**No •**
D.3.11.12	Homeopathic medicinal product?	**No •**
D.3.11.13	Another type of medicinal product?	**Yes •**
D.3.11.13.1	If 'another type of medicinal product' specify the type of medicinal product: **Saccharomyces is a probiotic yeast**	
D.3.12	Mode of action (*free text*[20]) **The probiotic yeast influence the commensal gut microbiota**	
D.3.13	Is it an IMP to be used in a first-in-human clinical trial?	**No •**
D.3.13.1	If 'Yes', are there risk factors identified, according to the guidance FIH?[21]	

**D.4**	**SOMATIC CELL THERAPY INVESTIGATIONAL MEDICINAL PRODUCT (NO GENETIC MODIFICATION)**	
D.4.1	Origin of cells	
D.4.1.1	Autologous	**No •**
D.4.1.2	Allogeneic	**No •**
D.4.1.3	Xenogeneic	**No •**
D.4.1.3.1	If 'Yes', specify the species of origin:	
D.4.2	Type of cells	
D.4.2.1	Stem cells	**No •**
D.4.2.2	Differentiated cells	**No •**
D.4.2.2.1	If 'Yes', specify the type (e.g. keratinocytes, fibroblasts, chondrocytes…):	
D.4.2.3	Others:	**No •**
D.4.2.3.1	If others, specify:	

**D.5**	**GENE THERAPY INVESTIGATIONAL MEDICINAL PRODUCTS**	
D.5.1	Gene(s) of interest:	
D.5.2	In vivo gene therapy:	**No •**
D.5.3	Ex vivo gene therapy:	**No •**
D.5.4	Type of gene transfer product	
D.5.4.1	Nucleic acid (e.g. plasmid):	**No •**
	If 'Yes', specify if:	
D.5.4.1.1	Naked:	**No •**
D.5.4.1.2	Complexed	**No •**
D.5.4.2	Viral vector:	**No •**
D.5.4.2.1	If 'Yes', specify the type: adenovirus, retrovirus, AAV, …:	
D.5.4.3	Others	**No •**
D.5.4.3.1	If others, specify:	
D.5.5	Genetically modified somatic cells:	**No •**
If 'Yes', specify the origin of the cells:		
D.5.5.1	Autologous:	**No •**
D.5.5.2	Allogeneic:	**No •**
D.5.5.3	Xenogeneic:	**No •**
D.5.5.3.1	If 'Yes', specify the species of origin:	
D.5.5.4	Specify type of cells (hematopoietic stem cells…):	

D.6 **TISSUE ENGINEERED PRODUCT**

The indication which determines that this is a Tissue Engineered Product as opposed to a Cell Therapy product is given in section E.1.1.

D.6.1	Origin of cells	
D.6.1.1	Autologous	**No •**
D.6.1.2	Allogeneic	**No •**
D.6.1.3	Xenogeneic	**No •**
D.6.1.3.1	If 'Yes', specify the species of origin:	

D.6.2	Type of cells	
D.6.2.1	Stem cells	**No •**
D.6.2.2	Differentiated cells	**No •**
D.6.2.2.1	If 'Yes', specify the type of cells(e.g. keratinocytes, fibroblasts, chondrocytes, …):	
D.6.2.3	Others:	**No •**
D.6.2.3.1	If others, specify:	

**D.7**	**PRODUCTS CONTAINING DEVICES (i.e. MEDICAL DEVICES, SCAFFOLDS ETC.)**	
D.7.1	Give a brief description of the device:	
D.7.2	What is the name of the device?	
D.7.3	Is the device implantable?	**No •**
D.7.4	Does this product contain:	
D.7.4.1	A medical device?	**No •**
D.7.4.1.1	Does this medical device have a CE mark?	**No •**
D.7.4.1.1.1	The notified body is:	
D.7.4.2	Bio-materials?	**No •**
D.7.4.3	Scaffolds?	**No •**
D.7.4.4	Matrices?	**No •**
D.7.4.5	Other?	**No •**
D.7.4.5.1	If other, specify:	

D.8 INFORMATION ON PLACEBO (if relevant; repeat as necessary)

D.8.1	Is there a placebo:	**Yes •**

D.8.2	This refers to placebo number:	**PL1**
D.8.3	Pharmaceutical form:	**Capsule**
D.8.4	Route of administration:	**Oral use**
D.8.5	Which IMP is it a placebo for? Specify IMP Number(s) from D.1.1	**PR1**
D.8.5.1	Composition, apart from the active substance(s):	
D.8.5.2	Is it otherwise identical to the IMP?	**No •**
D.8.5.2.1	If not, specify major ingredients: **microcrystalline cellulose, dibasic anhydrous calcium phosphate, silicon dioxide and magnesium stearate in order to maximally parallel the weight of other study products**	
D.8.5	Which IMP is it a placebo for? Specify IMP Number(s) from D.1.1	**PR2**
D.8.5.1	Composition, apart from the active substance(s):	
D.8.5.2	Is it otherwise identical to the IMP?	**No •**
D.8.5.2.1	If not, specify major ingredients: **microcrystalline cellulose, dibasic anhydrous calcium phosphate, silicon dioxide and magnesium stearate in order to maximally parallel the weight of other study products**	

D.9 SITE(S) WHERE THE QUALIFIED PERSON CERTIFIES BATCH RELEASE[22]

*This section is dedicated to **finished** IMPs, i.e. medicinal products randomised, packaged, labelled and certified for use in the clinical trial. If there is more than one site or more than one IMP is certified, use extra pages and*

give each IMP its number from section D.1.1 or D.8.2 In the case of multiple sites indicate the product certified by each site

D.9.1	Do not fill in section D.9.2 for an IMP that:<br>*Has a MA in the EU **and***<br>*Is sourced from the EU market **and***<br>*Is used in the trial without modification(e.g. not overencapsulated) **and***<br>*The packaging and labelling is carried out for local use only as per article 9.2. of the Directive 2005/28/EC (GCP Directive)*<br>If all these conditions are met tick **?**and list the number(s) of each IMP including placebo from sections D.1.1 and D.8.2 to which this applies<br>**PR1**<br>**PR2**<br>**PL1**

**D.9.2**	**Who is responsible in the Community for the certification of the finished IMPs?**	
	This site is responsible for certification of (list the number(s) of each IMP including placebo from sections D.1.1 and D.8.2):	
	please tick the appropriate box:	
D.9.2.1	Manufacturer	**Yes •**
D.9.2.2	Importer	**No •**
D.9.2.3	Name of the organisation:	**Metagenics**
D.9.2.4	Address:	
D.9.2.4.1	Street Address	**E. Vletinckstraat, 20**
D.9.2.4.2	Town/City	**Oostende**
D.9.2.4.3	Post Code	**8400**
D.9.2.4.4	Country	**Belgium**
D.9.2.5	Give the manufacturing authorisation number:	
D.9.2.5.1	If No authorisation, give the reasons:	

Where the product does not have a MA in the EU, but is supplied in bulk and final packaging and labelling for local use is carried out in accordance with article 9.2 of Directive 2005/28/EC (GCP Directive) then enter the site where the product was finally certified for release by the Qualified Person for use in the clinical trial at D.9.2 above.

E. GENERAL INFORMATION ON THE TRIAL

This section should be used to provide information about the aims, scope and design of the trial. When the protocol includes a sub-study in the MS concerned section E.2.3 should be completed providing information about the sub-study. To identify it check the sub-study box in the 'Objective of the trial' question below.

**E.1**	**MEDICAL CONDITION OR DISEASE UNDER INVESTIGATION**	
E.1.1	Specify the medical condition(s) to be investigated[23] (free text):	
	**English**	**Hospitalized patients suffering infection treated with the antibiotic amoxicillin-clavulanate**
E.1.1.1	Medical condition in easily understood language	
	**English**	**A person who suffers an infection that necessitate an hospitalisation and receive a treatment with the antibiotic amoxicillin-clavulanate.**
E.1.1.2	Therapeutic area	
	**Body processes [G] - Microbiological Phenomena [G06]**	
E.1.2	MedDRA version, system organ class, level, term and classification code[24]:	
	Version System Organ Class Classification Code Term Level	
E.1.3	Is any of the conditions being studied a rare disease[25]?	**No •**

**E.2**	**OBJECTIVE OF THE TRIAL**	
E.2.1	Main objective:	
	**English**	**Prevention of the colonisation of the gut microbiota with antibiotic resistant bacteria as Extended Spectrum Beta Lactamase (ESBL), AmpC β-lactamase (AmpC) and Carbapenemase Producing Enterobacteria (CPE), Vancomycin Resistant Enterococcus (VRE) or naturally resistant non-fermenting bacteria as Pseudomonas aeruginosa. Prevention of digestive discomfort due to the antibiotic treatment.**
E.2.2	Secondary objectives:	
	**English**	**Effect of the probiotic treatment on the length of stay, nosocomial infections or subsequent infection with antibiotic resistant bacteria.**
E.2.3	Is there a sub-study?	**No •**
E.2.3.1	If 'Yes', give the full title, date and version of each sub-study and their related objectives:	

**E.3**	**PRINCIPAL INCLUSION CRITERIA *(list the most important)***	
	**English**	**- Age over 18 years old.<br>- Infection treated with the antibiotic amoxicillin-clavulanate.<br>- Hospitalisation.<br>- Agree to give fecal sample.**

**E.4**	**PRINCIPAL EXCLUSION CRITERIA *(list the most important)***	
	**English**	**- Age under 18.<br>- Pregnancy, breastfeeding, or planning to become pregnant during the course of the trial.<br>- Average number of formed bowel movements more than 3 per day or les than 3 per week.<br>- Participation in a clinical research trial within 30 days prior to randomisation.<br>- Regular use of pro- and/or prebiotic products.<br>- Unstable medical conditions.<br>- History of chronic gastrointestinal disorders including irritable bowel syndrome, colitis, Crohn's disease or any other malabsorption or**

gastrointestinal disorder.
- Allergy or sensitivity to test product ingredients or antibiotics, allergy to any penicillin antibiotics.
- Patients receiving dialysis. Patient with central line.
- Deglutition abnormalities prohibiting or preventing a normal oral intake.
- Treatment with other antibiotics at the time of randomisation

**E.5**	**END POINT(S):**	
E.5.1	Primary End Point (repeat as necessary)[26]	
	**English**	**- Identification of antibiotic resistant bacteria (ESBL, AmpC, CPE, VRE, non-fermenting bacteria) in the stool sample.<br>- Evaluation of the digestive confort during and after the antibiotic treatment.**
E.5.1.1	Timepoint(s) of evaluation of this end point	
	**English**	**Stools are collected:<br>- at the time of inclusion in the trial<br>- at the end of the antibiotic treatment (usually correspond to the end of the hospital stay)<br>- at the end of the study treatment, one to three months after the release from hospital.**
E.5.2	Secondary End Point (repeat as necessary)	
	**English**	**- Evaluation of the incidence of infection due to antibiotic resistant bacteria<br>- Evaluation of the duration of the hospital stay.**
E.5.2.1	Timepoint(s) of evaluation of this end point	
	**English**	**During the hospital stay and after the release from hospital.**

**E.6**	**SCOPE OF THE TRIAL – Tick all boxes where applicable**	
E.6.1	Diagnosis	**Yes •**
E.6.2	Prophylaxis	**Yes •**
E.6.3	Therapy	**Yes •**
E.6.4	Safety	**Yes •**
E.6.5	Efficacy	**Yes •**
E.6.6	Pharmacokinetic	**No •**
E.6.7	Pharmacodynamic	**No •**
E.6.8	Bioequivalence	**No •**
E.6.9	Dose Response	**No •**
E.6.10	Pharmacogenetic	**No •**
E.6.11	Pharmacogenomic	**No •**
E.6.12	Pharmacoeconomic	**No •**
E.6.13	Others	**No •**
E.6.13.1	If others, specify:	

**E.7**	**TRIAL TYPE AND PHASE[27]**	
E.7.1	Human pharmacology (Phase I)	**No •**
Is it:		
E.7.1.1	First administration to humans	**No •**
E.7.1.2	Bioequivalence study	**No •**
E.7.1.3	Other:	**No •**
E.7.1.3.1	If other, please specify:	
E.7.2	Therapeutic exploratory (Phase II)	**No •**
E.7.3	Therapeutic confirmatory (Phase III)	**No •**

E.7.4	Therapeutic use(Phase IV)	**Yes •**

**E.8**	**DESIGN OF THE TRIAL**	
E.8.1	Controlled	**Yes •**
	If 'Yes', specify:	
E.8.1.1	Randomised:	**Yes •**
E.8.1.2	Open:	**No •**
E.8.1.3	Single blind:	**No •**
E.8.1.4	Double blind:	**Yes •**
E.8.1.5	Parallel group:	**No •**
E.8.1.6	Cross over:	**No •**
E.8.1.7	Other:	**No •**
E.8.1.7.1	If other specify:	
E.8.2	If controlled, specify the comparator:	
E.8.2.1	Other medicinal product(s)	**Yes •**
E.8.2.2	Placebo	**Yes •**
E.8.2.3	Other	**No •**
E.8.2.3.1	If 'Yes' to other, specify :	
E.8.2.4	Number of treatment arms in the trial	**3**
E.8.3	Single site in the Member State concerned (see also section G):	**Yes •**
E.8.4	Multiple sites in the Member State concerned(see also section G):	**No •**
E.8.4.1	Number of sites anticipated in Member State concerned	
E.8.5	Multiple Member States:	**No •**
E.8.5.1	Number of sites anticipated in the EEA:	
E.8.6	Trial involving sites outside the EEA:	
E.8.6.1	Trial being conducted both within and outside the EEA:	**No •**
E.8.6.2	Trial being conducted completely outside of the EEA:	**No •**
E.8.6.3	If E.8.6.1 or E.8.6.2 are Yes, specify the regions in which trial sites are planned:	
E.8.6.4	If E.8.6.1 or E.8.6.2 are Yes, specify the number of sites anticipated outside of the EEA:	
E.8.7	Trial having an independent data monitoring committee:	**Yes •**
E.8.8	Definition of the end of trial: If it is the last visit of the last subject, please enter "LVLS". If it is not LVLS provide the definition:	
	**English**	**The trial is ended when the last patient included has given the third stool sample or if the last patient refuse to give the third sample.**
E.8.9	Initial estimate of the duration of the trial[28] (years, months and days)	
E.8.9.1	In the Member State concerned	**years months days**
E.8.9.2	In all countries concerned by the trial	**years months days**
E.8.10	Proposed date of start of recruitment	
E.8.10.1	In the Member State concerned	
E.8.10.2	In any country	

F. POPULATION OF TRIAL SUBJECTS

**F.1**	**AGE RANGE**		
F.1.1	Are the trial subjects under 18?<br>If 'Yes', specify the estimated number of subjects planned in each age range for the whole trial:		**No •**
		Approx. No. of patients[29]	
F.1.1.1	In utero	()	**No •**
F.1.1.2	Preterm newborn infants (up to gestational age < 37 weeks)	()	**No •**
F.1.1.3	Newborns (0-27 days)	()	**No •**
F.1.1.4	Infants and toddlers (28 days - 23 months)	()	**No •**
F.1.1.5	Children (2-11 years)	()	**No •**
F.1.1.6	Adolescents (12-17 years)	()	**No •**
F.1.2	Adults (18-64 years)	(**120**)	**Yes •**
F.1.3	Elderly (>= 65 years)	(**120**)	**Yes •**

**F.2**	**GENDER**	
F.2.1	Female	**Yes •**
F.2.2	Male	**Yes •**

**F.3**	**GROUP OF TRIAL SUBJECTS**	
F.3.1	Healthy volunteers	**No •**
F.3.2	Patients	**Yes •**
F.3.3	Specific vulnerable populations	**No •**
F.3.3.1	Women of child bearing potential not using contraception	**No •**
F.3.3.2	Women of child bearing potential using contraception	**No •**
F.3.3.3	Pregnant women	**No •**
F.3.3.4	Nursing women	**No •**
F.3.3.5	Emergency situation	**No •**
F.3.3.6	Subjects incapable of giving consent personally	**Yes •**
F.3.3.6.1	If 'Yes', specify:<br>**English** **Older patient are more susceptible to be colonized and / or suffer infection with with antibiotic resistant bacteria. Older patient present more digestive discomfort dur to antibiotic treatment. They could have a benefit tof taking probiotics.**	
F.3.3.7	Others:	**No •**
F.3.3.7.1	If 'Yes', specify:	

**F.4**	**PLANNED NUMBER OF SUBJECTS TO BE INCLUDED:**	
F.4.1	In the member state	**120**
F.4.2	For a multinational trial**:**	
F.4.2.1	In the EEA	
F.4.2.2	In the whole clinical trial	

**F.5**	**PLANS FOR TREATMENT OR CARE AFTER THE SUBJECT HAS ENDED HIS/HER PARTICIPATION IN THE TRIAL. please specify** (free text):

G. CLINICAL TRIAL SITES/INVESTIGATORS IN THE MEMBER STATE CONCERNED BY THIS REQUEST

**G.1**	**CO-ORDINATING INVESTIGATOR (for multicentre trial) and principal investigator (for single centre trial)**	
G.1.1	Given name:	**Grégoire**
G.1.2	Middle name, if applicable:	**Jacques François Ghislain**
G.1.3	Family name:	**Wieërs**
G.1.4	Qualification (MD.........)	**MD PhD**
G.1.5	Professional address:	
G.1.5	Institution name	**Clinique Saint Pierre Ottignies**
G.1.5	Institution department	**Internal Medicine / infectiology**
G.1.5.1	Street address	**Avenue Reine Fabiola, 9**
G.1.5.2	Town/city	**Ottignies**
G.1.5.3	Post code	**1340**
G.1.5.4	Country	**Belgium**
G.1.6	Telephone number:	**0032 10 437493**
G.1.7	Fax number:	
G.1.8	E-mail:	**gregoire.wieers@cspo.be**

**G.2**	**PRINCIPAL INVESTIGATORS** ***(for multicentre trial ; where necessary, use additional forms)***
G.2.1	Given name:
G.2.2	Middle name, if applicable:
G.2.3	Family name:
G.2.4	Qualification (MD.........)
G.2.5	Professional address:
G.2.5	Institution name
G.2.5	Institution department
G.2.5.1	Street address
G.2.5.2	Town/city
G.2.5.3	Post code
G.2.5.4	Country
G.2.6	Telephone number:
G.2.7	Fax number:
G.2.8	E-mail:

**G.3**	**CENTRAL TECHNICAL FACILITIES TO BE USED IN THE CONDUCT OF THE TRIAL**<br><br>**Laboratory or other technical facility, in which the measurement or assessment of the main evaluation criteria are centralised** (repeat as needed for multiple organisations).	
G.3.1	Name of organisation:	
G.3.2	Department	
G.3.3	Name of contact person:	
G.3.3.1	Given name	
G.3.3.2	Middle name	
G.3.3.3	Family name	
G.3.4	Address:	
G.3.4.1	Street address	
G.3.4.2	Town/city	
G.3.4.3	Post code	
G.3.4.4	Country	
G.3.5	Telephone number:	
G.3.6	Fax number:	
G.3.7	E-mail:	
G.3.8	Enter the details of any duties subcontracted to this central technical facility in this trial	
G.3.8.1	Routine clinical pathology testing	**Yes ? No ? Not Answered ?**

G.3.8.2	Clinical chemistry	**Yes** ? **No** ? **Not Answered** ?
G.3.8.3	Clinical haematology	**Yes** ? **No** ? **Not Answered** ?
G.3.8.4	Clinical microbiology	**Yes** ? **No** ? **Not Answered** ?
G.3.8.5	Histopathology	**Yes** ? **No** ? **Not Answered** ?
G.3.8.6	Serology/ endocrinology	**Yes** ? **No** ? **Not Answered** ?
G.3.8.7	Analytical chemistry	**Yes** ? **No** ? **Not Answered** ?
G.3.8.8	ECG analysis/ review	**Yes** ? **No** ? **Not Answered** ?
G.3.8.9	Medical image analysis/ review - X-ray, MRI, ultrasound, etc.	**Yes** ? **No** ? **Not Answered** ?
G.3.8.10	Primary/ surrogate endpoint test	**Yes** ? **No** ? **Not Answered** ?
G.3.8.11	Other Duties subcontracted?	**Yes** ? **No** ? **Not Answered** ?
G.3.8.11.1	If 'Yes', specify the other duties	

**G.4**	**NETWORKS TO BE INVOLVED IN THE TRIAL (e.g. Paediatric Networks involved in the trial)**
G.4.1	Name of organisation:
G.4.2	Name of contact person:
G.4.2.1	Given name
G.4.2.2	Middle name
G.4.2.3	Family name
G.4.3	Address:
G.4.3.1	Street address
G.4.3.2	Town/city
G.4.3.3	Post code
G.4.3.4	Country
G.4.4	Telephone number:
G.4.5	Fax number:
G.4.6	E-mail:
G.4.7	Activities carried out by the network:

**G.5**	**ORGANISATIONS TO WHOM THE SPONSOR HAS TRANSFERRED TRIAL RELATED DUTIES AND FUNCTIONS**	
G.5.1	**Has the sponsor transferred any major or all the sponsor's trial related duties and functions to another organisation or third party?**	**No** •
Repeat as necessary for multiple organisations:		
G.5.1.1	Organisation name:	
G.5.1.2	Organisation department	
G.5.1.3	Name of contact person :	
G.5.1.3.1	Given name	
G.5.1.3.2	Middle name	
G.5.1.3.3	Family name	
G.5.1.4	Address:	
G.5.1.4.1	Street address	
G.5.1.4.2	Town/city	
G.5.1.4.3	Post code	
G.5.1.4.4	Country	
G.5.1.5	Telephone number:	
G.5.1.6	Fax number:	
G.5.1.7	E-mail:	
G.5.1.8	All tasks of the sponsor	**Yes** ? **No** ? **Not Answered** ?
G.5.1.9	Monitoring	**Yes** ? **No** ? **Not Answered** ?
G.5.1.10	Regulatory (e.g. preparation of applications to CA and ethics committee)	**Yes** ? **No** ? **Not Answered** ?
G.5.1.11	Investigator recruitment	**Yes** ? **No** ? **Not Answered** ?
G.5.1.12	IVRS[30] – treatment randomisation	**Yes** ? **No** ? **Not Answered** ?
G.5.1.13	Data management	**Yes** ? **No** ? **Not Answered** ?
G.5.1.14	E-data capture	**Yes** ? **No** ? **Not Answered** ?
G.5.1.15	SUSAR reporting	**Yes** ? **No** ? **Not Answered** ?
G.5.1.16	Quality assurance auditing	**Yes** ? **No** ? **Not Answered** ?

G.5.1.17	Statistical analysis	**Yes** ? **No** ? **Not Answered** ?
G.5.1.18	Medical writing	**Yes** ? **No** ? **Not Answered** ?
G.5.1.19	Other duties subcontracted?	**Yes** ? **No** ? **Not Answered** ?
G.5.1.19.1	If 'Yes' to other, please specify:	

H. COMPETENT AUTHORITY / ETHICS COMMITTEE IN THE MEMBER STATE CONCERNED BY THIS REQUEST

**H.1**	**TYPE OF APPLICATION**	
If this application is addressed to the Competent Authority, please tick the Ethics Committee box and give information on the Ethics committee concerned. If this application is addressed to the Ethics Committee, please tick the Competent Authority box and give the information on the Competent Authority concerned.		
H.1.1	Competent Authority	**No •**
H.1.2	Ethics Committee	**Yes •**

**H.2**	**INFORMATION ON ETHICS COMMITTEE**	
H.2.1	Name:	**Comité Hospitalo-Facultaire Universitaire de Liège 707**
H.2.2	Address	
H.2.2.1	Street address	**Domaine Universitaire du Sart Tilman**
H.2.2.2	Town/city	**Liège**
H.2.2.3	Post code	**4000**
H.2.2.4	Country	**Belgium**
H.2.3	Date of submission:	**2017-05-24**

**H.3**	**OPINION**	
H.3.1	To be requested	**No •**
H.3.2	Pending	**No •**
H.3.3	Given	**Yes •**
	If 'Given', specify:	
H.3.3.1	Date of opinion:	**2017-06-07**
H.3.3.2	Opinion favourable	**Yes •**
H.3.3.3	Opinion not favourable	**No •**
	If not favourable, give:	
H.3.3.3.1	The reasons	
H.3.3.3.2	The eventual anticipated date of resubmission:	

I. SIGNATURE OF THE APPLICANT IN THE MEMBER STATE

**I.1**	I hereby confirm that /confirm on behalf of the sponsor (delete which is not applicable) that:<br>• the information provided is complete;<br>• the attached documents contain an accurate account of the information available;<br>• the clinical trial will be conducted in accordance with the protocol; and<br>• the clinical trial will be conducted, and SUSARs and result-related information will be reported, in accordance with the applicable legislation.

**I.2**	**APPLICANT OF THE REQUEST FOR THE COMPETENT AUTHORITY** (as stated in section C.1):
I.2.1	Date:
I.2.2	Signature[31]:
I.2.3	Print name:

**I.3**	**APPLICANT OF THE REQUEST FOR THE ETHICS COMMITTEE** (as stated in section C.2):
I.3.1	Date:
I.3.2	Signature[32]:
I.3.3	Print name:

ENDNOTES

[1] Any translation of the protocol should be assigned the same date and version as those in the original document.
[2] International Standard Randomised Controlled Trial Number. Sponsors may wish to use an International Standardised Random Controlled Trial Number (ISRCTN) to identify their trial in addition to the EudraCT number; for instance if their trial is part of a multinational trial with sites outside the Community. They can obtain the number and guidance from the Current Controlled Trials website http://www.controlled-trials.com/isrctn to which there is a link from the EudraCT database website http://eudract.ema.europa.eu. When available they should provide it in Section A.6 of the application form.
[3] US National Clinical Trial (NCT) Numbers required on the FDA clinical trial application form.
[4] For a resubmission following previous withdrawal of an application or unfavourable opinion of an ethics committee, or previous withdrawal of an application or refusal of a request by the competent authority, enter a letter in the sequence, A for first resubmission, B for second, C for third et seq.
[5] In accordance with Article 19 of Directive 2001/20/EC.
[6] The contact point should give functional information rather than details of one "person", in order to avoid the need for update and maintenance of these contact details.
[7] This requires a EudraLink account. (See https://eudract.ema.europa.eu/document.html for details)
[8] According to national legislation.
[9] Available from the Summary of Product Characteristics (SmPC)
[10] According to the Community register on orphan medicinal products (Regulation (EC) n° 141/2000): http://ec.europa.eu/enterprise/pharmaceuticals/register/index.htm
[11] Committee for Medicinal Products for Human Use of the European Medicines Agency
[12] To be provided only when there is No trade name. This is the name routinely used by a sponsor to identify the IMP in the CT documentation (protocol, IB…).
[13] To be provided only when there is No trade name. This is a code designated by the sponsor which represents the name routinely used by the sponsor to identify the product in the CT documentation. For example, a code may be used for combinations of drugs or drugs and devices.
[14] Available from the Summary of Product Characteristics (SmPC).
[15] Chemical Abstracts Service.
[16] Complete also section D.4 Cell therapy as defined in Annex 1 part IV of Directive 2001/83/EC as amended.
[17] Complete also section D.5 Gene Therapy as defined in Annex 1 part IV of Directive 2001/83/EC as amended.
[18] Complete also section D.6 - Tissue Engineered Product as defined in Article 2(1)(b) of Regulation1394/2007/EC.
[19] Complete also section D.7
[20] The mode of action should briefly describe the chemical, biochemical, immunological or biological means the IMP uses to effect its pharmaceutical action.
[21] Guideline on strategies to identify and mitigate risks for first-in-human clinical trials with investigational medicinal products. EMEA/CHMP/SWP/28367/2007 19 July 2007
[22] In accordance with paragraph 38 of Annex 13 of Volume 4 of the Rules Governing Medical Products in the European Union.
[23] In the case of healthy volunteer trials, the intended indication for the product under development should be provided.
[24] Applicants are encouraged to provide the MedDRA lower level term if applicable and classification code. These can be accessed from the EMEA EudraCT website (http://eudract.ema.europa.eu/).
[25] Points to consider on the calculation and reporting of the prevalence of a condition for Orphan drug designation: COM/436/01 (http://www.ema.europa.eu/htms/human/orphans/intro.htm).
[26] The protocol will usually identify a single primary end point but there may be a co-primary end point in some cases and/or a number of secondary end points.
[27] The descriptions of the trial types provided are those recommended in preference to Phases. See page 5 of Community guideline CPMP/ICH/291/95. The development of a new indication after initial approval of a medicine should be considered as a new development plan.
[28] From the first inclusion until the last visit of the last subject.
[29] These numbers will be initial estimates. Applicants will not be required to update this information nor do they constitute an authorisation or restriction on the inclusion of these numbers of patients in the trial. The numbers of subjects whose inclusion is authorised are those set out in the authorised version of the protocol, or subsequent authorised amendments.
[30] Interactive Voice Response System: commonly used for randomisation of treatment and controlling the shipment of stock of product.
[31] On an application to the Competent Authority only, the applicant to the Competent Authority needs to sign.

[32] On an application to the Ethics Committee only, the applicant to the Ethics Committee needs to sign.
